# Rocket (*Eruca vesicaria* (L.) Cav.) vs. Copper: The Dose Makes the Poison?

**DOI:** 10.3390/molecules27030711

**Published:** 2022-01-21

**Authors:** Mario Nikola Mužek, Franko Burčul, Dario Omanović, Azra Đulović, Sandra Svilović, Ivica Blažević

**Affiliations:** 1 Department of Inorganic Technology, Faculty of Chemistry and Technology, University of Split, Ruđera Boškovića 35, 21000 Split, Croatia; 2 Department of Analytical Chemistry, Faculty of Chemistry and Technology, University of Split, Ruđera Boškovića 35, 21000 Split, Croatia; 3 Laboratory for Physical Chemistry of Traces, Division for Marine and Environmental Research, Ruđer Bošković Institute, Bijenička cesta 54, 10000 Zagreb, Croatia; omanovic@irb.hr; 4 Department of Organic Chemistry, Faculty of Chemistry and Technology, University of Split, Ruđera Boškovića 35, 21000 Split, Croatia; azra@ktf-split.hr (A.Đ.); blazevic@ktf-split.hr (I.B.); 5 Department of Chemical Engineering, Faculty of Chemistry and Technology, University of Split, Ruđera Boškovića 35, 21000 Split, Croatia; sandra@ktf-split.hr

**Keywords:** Rocket (*Eruca vesicaria* (L.) Cav.), copper, glucosinolates, phytoremediation

## Abstract

The effects of copper addition, from various adsorbents, on the accumulation ability and glucosinolate content of cultivated rocket were studied. Different adsorbents (zeolite NaX, egg shells, substrate, fly ash) were treated with copper(II) solution with an adsorption efficiency of 98.36, 96.67, 51.82 and 39.13%, respectively. The lowest copper content and the highest total glucosinolate content (44.37 μg/g DW and 4269.31 µg/g DW, respectively) were detected in the rocket grown in the substrate with the addition of a substrate spiked with copper(II) ions. Rocket grown in the fly ash-substrate mixture showed an increase in copper content (84.98 μg/g DW) and the lowest total glucosinolate content (2545.71 µg/g DW). On the other hand, when using the egg shells-substrate mixture, the rocket copper content increased (113.34 μg/g DW) along with the total GSLs content (3780.03 µg/g DW), indicating the influence of an adsorbent type in addition to the copper uptake. The highest copper content of 498.56 μg/g DW was detected in the rocket watered with copper(II) solution with a notable decrease in the glucosinolate content, i.e., 2699.29 µg/g DW. According to these results rocket can be considered as a copper accumulator plant.

## 1. Introduction

Metal pollutants are discharged in large amounts in the environment. A fundamental problem of metals as pollutants is that they do not degrade [1]. Some of these metals, like Cd, Hg, or Pb are non-nutrients, and so are extremely toxic for plants. Others, like Co, Cu, Fe, Mn, Mo, Ni, and Zn, are classified as essential [2,3]. Nevertheless, the phrase “The dose makes the poison”, could be applied for essential elements as well.

Copper is needed for the wellbeing of both plants and animals, having an important role in numerous physiological functions such as photosynthesis, respiration, C and N metabolism, and protection against oxidative stress, etc. [4]. It represents one of the most used industrial metals in the world, but its application does not end with industry. Copper compounds are used in agriculture, biology, chemistry, etc. The presence of large amounts of copper in the environment causes a severe problem in environmental protection. It can enter the food chain and be absorbed by all living organisms causing acute health disorders [5]. A considerable number of studies have explored various methods for metal removal from the environment [6]. Those studies are often based on the adsorption of metals on various adsorbents in order to clean the environment and reduce waste volume. Soil contamination causes deep concerns, thus it is important to carefully dispose of the adsorbents saturated with metal ions and, if possible, reduce the waste volume even further [7]. Taking the increasing demand for water in agriculture into account, there is a possibility of using the treated wastewater, that can contain heavy metals, which can pose a danger due to the accumulating ability of plants used in the human diet [8].

Owing to nature’s self-preservation capability, some plants, including a number of plants from the Brassicaceae family, possess a way to extract toxic elements through a process known as phytoremediation. Phytoremediation has been known since the 1950s, although the term is from 1991 [9]. It is a cost effective, eco-friendly bioremediation process that uses plants for soil or water cleaning by removing, uptaking or rendering contaminants harmless thanks to the ability of some plants to transport and accumulate contaminants. The mechanism of phytoremediation and its efficiency depend on various factors, including plant species, nature of the contaminant, bioavailability, etc. [10,11].

Within the Brassicaceae family, several species are able to hyperaccumulate heavy metals [12]. The accumulation of such high levels of toxic metals was proposed to act as an elemental defense against herbivores that are unable to detoxify the metals [13]. The Brassicaceae family is also, without exception, strikingly chemo-characterized by the presence of thiosaccharidic specialized metabolites-glucosinolates (GSLs), which act as defense compounds readily activated by enzymes upon plant damage forming toxic hydrolysis products, mostly isothiocyanates [14,15]. Various biological activities of isothiocyanates have been demonstrated previously with imperative anticarcinogenic properties by suppressing various critical hallmarks of cancer (cellular proliferation, apoptosis, metastasis, etc.) in vitro as well as in vivo [16]. In general, heavy metal accumulation can influence the GSL content in various ways: by decreasing it [12], increasing it [17], or resulting in no effect [18,19]. Copper excess can be highly phytotoxic as it can generate hydroxyl radicals causing damage to lipids, proteins and DNA [20], interfere with iron homeostasis [21], reduce plant biomass, inhibit root growth, and induce chlorosis, bronzing and necrosis. Morelli and Scarano demonstrated that prolonged copper exposure induced membrane damage, as well as synthesis of gluthathione and its derived peptides as a first line of defense against copper induced reactive oxygen species formation [22]. The plant can manage copper excess by chelation with thiol rich compounds, such as cysteine-rich proteins, or phytochelatins synthesized from glutathione [23].

Rocket (also known as arugula, rucola, and roquette, *Eruca vesicaria* (L.) Cav.) is a known crop from the Brassicaceae family. It is appreciated for its tolerance and adaptability to unfavorable environmental conditions, including resistance to powdery mildew, stem rot and salt [24]. Zhi et al. reported that rocket seed germination is quite tolerant to copper for tested concentrations (0.3–1.2 mM), while lower concentrations (<0.7 mM) even increased the seed germination process [23]. Rocket is known for producing some quite unique GSLs, but three most abundant ones are 4-mercaptobutyl GSL (glucosativin), 4-(methylsulfanyl)butyl GSL (glucoerucin), and 4-(methylsulfinyl)butyl GSL (glucoraphanin) [15,25]. It was shown that metals such as As, Cd, Pb and Zn can induce GSLs’ production in rocket [26]. 

The presence of heavy metals in the environment and in the food chain is mainly undesirable, but in the case of phytoremediation the bigger picture should be taken into account. Although the high amount of copper, and other bioelements, could cause health disorders, they are also important for human health. So, it is important to elucidate how the various sources of metals, added to soil in which the plant is cultivated, affect the amount of metals in the plant, as well as on the production of plant’s secondary metabolites, important for human nutrition and health. So, the phytoremediation with plants used in nutrition could be linked not only with waste disposal minimization but to the enrichment of those plants with compounds useful for humans [27].

For all the reasons mentioned above the aims of this work were to study the cultivation ability of rocket in a substrate spiked with copper(II) ions adsorbed on various adsorbents, its accumulation ability, and its response to the induced abiotic stress by variations in the GSL content.

## 2. Results and Discussion

The amount of copper(II) ions adsorbed in the adsorbents which were used as a copper source is given in Table 1. Each adsorbent (copper source) was used as an additive to the substrate in which the rocket was grown to evaluate the plant’s accumulating ability.

Results in Table 1 show that the highest amount of copper, from the aqueous copper(II) sulfate solution, was adsorbed by zeolite NaX and egg shells (24.85 and 24.40 mg/g respectively). The substrate adsorbed a lower amount of copper(II) ions (13.09 mg/g), and the lowest amount of copper(II) ions was adsorbed by fly ash (9.85 mg/g). The high adsorption efficiency of NaX and egg shells, and the mediocre adsorption efficiency of the substrate and fly ash were in accordance with the literature [7,28,29,30,31].

**Figure 1:**
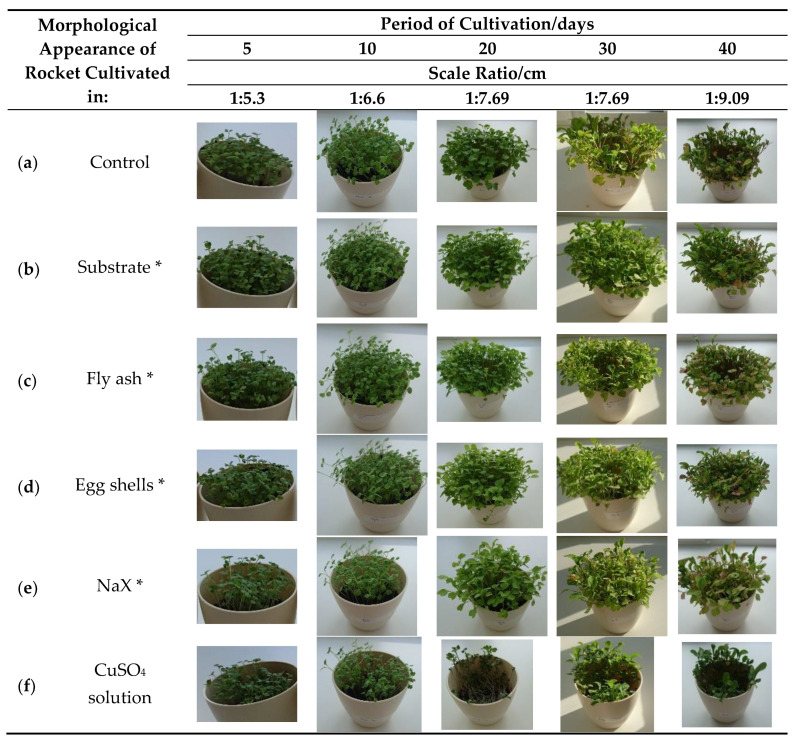
Morphological appearance of the rocket (*Eruca vesicaria* (L.) Cav.) cultivated in differently prepared substrate. Control—plant cultivated in pure Compo Sana substrate and watered with tap water; * Compo Sana substrate mixed with various adsorbents cf. Table 1 and watered with tap water; CuSO_4_ solution—plant cultivated in pure Compo Sana substrate and watered with copper(II) sulfate solution.

After cultivation, the lowest amount of copper in the plant was found in the control sample (12.19 μg/g dry weight (DW)), followed by the plant cultivated in the substrate with the addition of various adsorbents saturated with copper(II) ions: substrate (44.37 μg/g DW), fly ash (84.98 µg/g DW), egg shells (113.34 µg/g DW), and NaX (156.19 µg/g DW) (Table 2). The copper content in all the cultivated plants, besides in the control sample, exceeded the normal concentration range in plants (1–30 μg/g DW) [4]. There were no visible harmful effects on the plant growth and development for samples given in Figure 1b–d in comparison to the control sample (Figure 1a). When the plant was cultivated in the substrate with NaX or watered with the copper(II) solution, some negative effects on the plant development could be observed after fifth and tenth day of cultivation (Figure 1e(5,10)). The influence of copper(II) solution on the plant was especially visible after the twentieth day of cultivation (Figure 1f(20)). However, it is noteworthy to point out that the plant won over and continued with development, as can be seen after 30 and 40 days of cultivation (Figure 1f(30,40)). This effect was anticipated as it is proven that salinity, especially chloride and sulfate salinity, can result in reduced plant biomass [32]. The highest copper content in the rocket was detected in samples watered with copper(II) solution (498.56 μg/g DW). The lowest copper content was found in the plant grown in the substrate mixed with a substrate saturated with copper(II) ions, which can be attributed to two reasons: (i) a relatively low amount of copper(II) ions adsorbed in the substrate and (ii) its adsorption form (strength) that affects metal availability [31]. An interesting result was found for samples cultivated with the zeolite NaX addition. According to the literature and our previous study, it was expected that copper(II) ions adsorbed in zeolite NaX would not be available for plant uptake in high amount. Given the above reason, the plants cultivated in substrate with the addition of spiked zeolite NaX should not contain a high amount of copper [7,33]. However, in this study the second highest amount of copper in rocket was detected in this sample. These findings could be explained by the joint effect of pH (6.98) and the influence of the presence of humic (HA) and fulvic acid (FA) on copper(II) adsorption. The presence of humic and fulvic acid is reported to decrease copper(II) adsorption in ZSM-5 zeolite at higher pH values (>6) due to the formation of soluble HA/FA-Cu(II) complexes in the solution [34]. However, it is interesting to notice that when the copper content increased in the rocket grown in different substrate types, the content of Cd, Mn, and Zn decreased (Table 2). Some available or exchangeable metals can disturb the accumulation of Mn, Zn, and Cd [35]. Lombi et al. reported that the presence of copper in high amounts can seriously limit the potential for the phytoextraction of Zn and Cd in several *Brassica* species [36]. 

According to the literature data, copper hyperaccumulators are plants that accumulate >1000 µg/g without showing signs of phytotoxicity, and copper accumulators are plants that accumulate >100 µg/g [37]. Hyperaccumulation of copper was later revised downwards to >300 µg/g [38]. Since the rocket contained copper in amounts over 100 µg/g, when cultivated in substrate with the addition of egg shells and with the addition of zeolite NaX, as well as when watered with copper(II) solution (Table 2), it can be suggested that the rocket is a copper accumulator plant.

**Figure 2:**
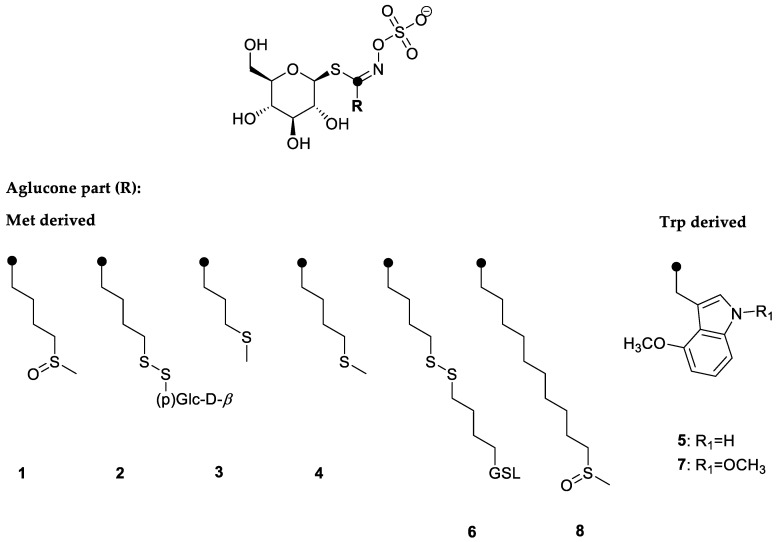
Structures of the glucosinolates identified in the rocket: **1**—4-(methylsulfinyl)butyl GSL (glucoraphanin); **2**—4-(*β*-d-glucopyranosyldisulfanyl)butyl GSL (diglucothiobeinin); **3**—3-(methylsulfanyl)propyl GSL (glucoibervirin); **4**—4-(methylsulfanyl)butyl GSL (glucoerucin); **5**—4-methoxyindol-3-ylmethyl GSL (4-methoxyglucobrassicin); **6**—dimeric 4-mercaptobutyl GSL; **7**—1,4-dimethoxyindol-3-ylmethyl GSL (1,4-dimethoxyglucobrassicin); **8**—10-(methylsulfinyl)decyl GSL (glucocamelinin). Chromatograms were given in Figure S1.

In response to the copper stress, the content of GSLs was measured. GSLs identified in the rocket were aliphatic ones that derived from methionine biosynthesis, i.e., glucoraphanin (**1**), 4-(*β*-d-glucopyranosyldisulfanyl)butyl GSL (diglucothiobeinin, (**2**), glucoibervirin (**3**), glucoerucin (**4**), dimeric 4-mercaptobutyl GSL (**6**), and glucocamelinin (**8**), while 4-methoxyglucobrassicin (**5**) and 1,4-dimethoxyglucobrassicin (**7**) derived from tryptophane biosynthesis. The structures are given in Figure 2. In general, the main GSLs in the rocket were aliphatic ones, i.e., **6**, **4**, **8**, **1**, and **2** in descending order, according to their content (Table 3). It was noticed that copper accumulation influenced the content of GSLs. This was especially observed in the case of the largest content of copper accumulated by the plant, where the total GSL content decreased to 2699.29 µg/g DW, similar to our previous report for the garden candytuft [7]. When investigating the influence of copper on Chinese cabbage, Shabaz et al. suggested that the impact on the GSL metabolism might be due to the interference of copper with sulfur and nitrogen metabolism, although it remained unclear to what extent and how it was regulated [39]. Recently, Aghajanzadeh et al., investigating the same plant, reported that when exposed to the elevated copper(II) ion concentration, no direct connection between the GSL and sulfur/nitrogen levels was observable (either in the roots or shoots). It was also concluded that the impact of sulfur on the GSL content differs between plant species, their developmental stage as well as between various organs [40]. In the case of fly ash addition, the GSL content decreased to a similar amount of 2545.71 µg/g DW. According to Khan et al. the addition of fly ash to the soil caused significant reductions in the nitrogen content [41]. This indicates that next to the copper amount, fly ash also has a negative effect on the GSL content. Contrary, the moderately high copper content in plant even increased the content of GSLs. Under metal stress, plants can produce higher amount of primary and secondary metabolites up to a certain copper content, beyond which a decrease of its content was observed [42].

These sulfur metabolites are the main defense of the Brassicaceae and often involve metabolic costs for plants. Boyd suggested balance between organic and elemental defenses in hyperaccumulators, meaning that the higher metal concentration in plants could result in producing lower amounts of defensive secondary metabolites [43]. According to the literature, some hyperaccumulator species have lower levels of GSLs in regard to congeneric nonaccumulators [18]. When rocket was watered only with copper(II) solution, or when it was cultivated in the substrate with the addition of NaX or fly ash, both spiked with copper(II) ions, the higher copper content resulted in lower amounts of GSLs. The only increase in the total amounts of GSLs was noticed when rocket was cultivated in the substrate with the addition of egg shells or extra added substrate, respectively. Both are known to possess essential nutrients for plant growing, and showed moderately higher copper contents. Egg shells contain calcium in the form of calcium carbonate and trace minerals, and its addition to the soil increases the nutritional intake of plants and enhances its growth [44].

The impact of sulfate salinity on GSL content is cation dependent, as well as to the added amount of the salt. Aghajanzadeh et al. investigated the impact of Na_2_SO_4_ and K_2_SO_4_ on the GSL content of *Brassica rapa*. While the addition of K_2_SO_4_ did not affect the total GSL content of both root and shoot, the addition of Na_2_SO_4_ enhanced total GSL content by increasing it [32].

## 3. Materials and Methods

### 3.1. General

Seeds of rocket (*Eruca vesicaria* (L.) Cav.) were obtained commercially (Bauhaus, Zagreb, Croatia). Copper sulfate pentahydrate (CuSO_4_∙5H_2_O) was obtained from Kemika, p.a; substrate (COMPO SANA) was manufactured by Compo GmbH (Compo GmbH, Münster, Germany) [7]. Zeolite NaX, DEAE-Sephadex A-25 anion-exchange resin, sulfatase (type H-1 from *Helix pomatia*) and sinigrin, were obtained from Sigma Aldrich (St. Louis, MO, USA), while glucoraphanin (**1**); glucoerucin (**4**); 4-methoxyglucobrassicin (**5**); and glucocamelinin (**8**) were obtained from Phytoplan (Heidelberg, Germany). Fly ash was collected from the Plomin2 power plant (Plomin, Croatia). According to the ASTM C618 it was classified as a type F and its detailed chemical analysis is given in previous work [45]. All other chemicals and reagents were of analytical grade.

The following instruments were used for analyses: UV/Vis spectrophotometer (Lambda EZ201, Perkin Elmer, Waltham, MA, USA), UHPLC-DAD-MS/MS (Ultimate 3000RS equipped with DAD and TSQ Quantis MS/MS detectors, Thermo Fischer Scientific, Waltham, MA, USA) using Hypersil GOLD C18 column (3.0 µm, 3.0 × 100 mm, Thermo Fischer Scientific, Waltham, MA, USA). HR ICP-MS (Thermo Finnigan, Bremen, Germany) was used for analysis of heavy metals. Quality control of HR ICP-MS measurement was checked by the determination of elements concentration in “River Water Reference Material for Trace Metals” (SLRS-5, National Research Council Canada). For most elements, a good agreement with the certified data was obtained.

### 3.2. Preparation of Adsorbents and Adsorption Studies

Zeolite NaX (particle size < 45 μm), fly ash (particle size < 45 μm), egg shells (particle size < 125 μm), and Compo Sana substrate (particle size < 500 μm), were used for copper adsorption.

The initial solution containing copper(II) ions (7.95 mM), used for adsorption and for watering, was prepared by dissolving CuSO_4_∙5H_2_O in distilled water. Adsorption of the copper(II) ions on adsorbents used was explained in detail in the previous work [7].

### 3.3. Metal Accumulation Studies

As a control sample, the rocket was planted in the untreated substrate and watered only with tap water. After copper adsorption and filtration through Whatman 42 filter paper, adsorbents were mixed with substrate. The rocket seeds were planted in flowerpots which were filled with a mixture of substrate (35 g) and adsorbents saturated with copper (10 g). The planted rocket was watered exclusively with tap water. Additionally, rocket was planted in pure Compo Sana substrate and watered with 590 cm^3^ of copper(II) solution in total. All the samples were watered with tap water or the copper(II) solution every two or three days when necessary. Shoots emerged after 30 h. Average shoot growth rate was 5 mm/day (monitored for first 16 days). After forty (40) days of cultivation, the rocket was harvested. Plant/root height ratio was 5:3 (total height plant + root was 16 cm). Three (3) parallel pots were used for each treatment.

### 3.4. Samples Preparation and Analysis

Samples of harvested rocket were prepared as previously reported [7,46]. For metal analysis, 1 g of dried plant material was used, and for isolation and analysis of GSLs, 100 mg was used.

The pH of substrate of the control sample after harvesting was pH = 6.85, but this value changed depending on the adsorbent addition as follows: substrate with zeolite NaX addition (pH = 6.98); substrate with extra substrate addition (pH = 6.72); substrate with egg shells addition (pH = 7.04), substrate with fly ash addition (pH = 6.55). The pH of substrate where the rocket was watered with copper(II) solution was pH = 5.86.

Quantification of copper(II) concentration in solutions before and after adsorption on various adsorbents was performed using UV/Vis at the wavelength of 810 nm with a calibration curve ranging from 0.60 to 21 mM. HR ICP-MS was used for the analysis of heavy metals in the samples obtained from the rocket after cultivation. UHPLC-DAD-MS/MS was used for analysis of GSLs by their dGSL counterparts. Quantification of dGSLs was performed using an external calibration curve of pure desulfosinigrin (ranging from 13.56 to 542.50 µM). For each individual dGSL response factors (RPF) were taken in accordance to the literature: RPF 1.07 for **1**, 1.04 for **4**, 0.25 **5** [47], 0.8 for **3** [48], 0.2 for **7** [49], while for **2**, **6**, and **8** arbitrary RPF of 1 was used.

### 3.5. Statistical Analysis

Analysis of variance (one-way ANOVA) was used to assess the statistical difference between data reported in Table 1, followed by a least significant difference test to evaluate differences between sets of mean values at significance level set at *p* < 0.05. Analyses were carried out using Statgraphics Centurion-Ver.16.1.11 (StatPoint Technologies, Inc., Warrenton, VA, USA) [50].

## 4. Conclusions

Adsorption on various adsorbent materials is a successful technique for the removal of a wide range of contaminants, including metal ions. Such materials are usually managed by disposing them in a landfill after they are no longer useful, which may lead to secondary pollution problems. The rocket’s appearance showed some differences during the cultivation period when grown in the combination of substrate and various adsorbents spiked with copper(II) ions. Egg shells, known as natural fertilizers, have shown to be an efficient bio-sorbent material of copper(II) ions. The rocket grown on the substrate with the addition of egg shells spiked with copper(II) ions showed a higher intake of copper, as well as an increased content of GSLs when compared to the control. On the other hand, when the plant was watered with copper(II) solution the influence was more conspicuous. The highest copper content in the rocket resulted in a significantly lower amount of total GSLs. Furthermore, the GSL’s total content decreased with the increase of the copper amount taken up by the plant, with the exception of fly ash. This suggests that, next to the adsorption efficiency, selection of adsorbent material is important as well, although it is not clear to what extent. Additionally, these results may infer the possible balance between organic and elemental defenses in (hyper)accumulators.

Generally, the obtained results indicated that the rocket can be considered as a (hyper)accumulator plant, when exposed to higher levels of copper. Further research is needed to fully elucidate the influence of copper uptake on the GSL content in the rocket, as well as its mechanism of accumulation along with the type of adsorbent/additive used as the copper source. Taking into the account that the rocket has the ability to uptake a significant amount of copper, and the fact that this plant is fast growing, suggests the potential application in phytoremediation.

## Figures and Tables

**Table 1:** Experimental data obtained after 10 days of adsorption process.

Adsorbent/Concentration	Zeolite NaX	Egg Shells	Substrate	Fly Ash
*c*_10_ (mmol/dm^3^)	0.13	0.27	3.83	4.84
*q*_10_ (mmol/g)	0.39	0.38	0.21	0.16
*q*_10_ (mg/g)	24.85	24.40	13.09	9.85
Adsorption efficiency (%)	98.36	96.67	51.82	39.13

*c*_10_ is the remaining concentration of copper(II) ions in the solution; *q*_10_ is the amount of the copper retained on the adsorbent.

**Table 2:** Heavy metal content in the cultivated rocket (whole plant).

	Heavy Metal (μg/g DW)							
	Cu	Cd	Mo	Mn	Fe	Co	Ni	Zn
Compo Sana substrate	10.19 ± 0.02 ^a^	0.05 ± 0.00 ^a^	0.01 ± 0.00 ^a^	19.29 ± 0.04 ^a^	263.31 ± 0.85 ^a^	0.13 ± 0.00 ^a^	0.92 ± 0.00 ^a^	15.41 ± 0.02 ^a^
Control	12.19 ± 0.16 ^b^	0.43 ± 0.00 ^b^	0.25 ± 0.00 ^b^	227.48 ± 1.99 ^b^	49.19 ± 0.84 ^b^	0.04 ± 0.00 ^b^	1.66 ± 0.04 ^b^	239.96 ± 2.21 ^b^
Substrate *	44.37 ± 0.49 ^c^	0.16 ± 0.00 ^c^	0.18 ± 0.00 ^c^	88.35 ± 0.77 ^c^	45.23 ± 0.28 ^c^	0.03 ± 0.00 ^c^	0.62 ± 0.00 ^c^	123.79 ± 1.33 ^c^
Fly ash *	84.98 ± 0.39 ^d^	0.14 ± 0.00 ^d^	0.48 ± 0.02 ^d^	79.13 ± 0.34 ^d^	82.49 ± 0.72 ^d^	0.12 ± 0.00 ^d^	1.82 ± 0.00 ^d^	108.86 ± 1.18 ^d^
Egg shells *	113.34 ± 0.85 ^e^	0.11 ± 0.00 ^e^	0.23 ± 0.01 ^e^	66.45 ± 0.83 ^e^	29.88 ± 0.29 ^e^	0.03 ± 0.00 ^c^	1.16 ± 0.01 ^e^	74.62 ± 0.38 ^e^
NaX *	156.19 ± 1.54 ^f^	0.07 ± 0.00 ^f^	0.26 ± 0.01 ^b^	58.86 ± 1.05 ^f^	72.45 ± 0.34 ^f^	0.05 ± 0.00 ^e^	0.88 ± 0.01 ^f^	78.99 ± 0.64 ^f^
CuSO_4_ solution	498.56 ± 1.77 ^g^	0.60 ± 0.00 ^g^	0.31 ± 0.00 ^f^	185.68 ± 0.41 ^g^	52.46 ± 0.31 ^g^	0.04 ± 0.00 ^b^	0.70 ± 0.00 ^g^	45.03 ± 0.63 ^g^

* Control—plant cultivated in pure Compo Sana substrate and watered with tap water; * Compo Sana substrate mixed with various adsorbents cf. Table 1 and watered with tap water; CuSO_4_ solution—plant cultivated in pure Compo Sana substrate and watered with copper(II) sulfate solution; DW—dry weight. Results are presented as mean ± SD (n = 3). Different superscript letters ^a–g^ in the same column denote statistically significant difference (*p* < 0.05) between samples.

**Table 3:** Glucosinolate content in the cultivated rocket (whole plant).

	Glucosinolates (μg/g DW)								
	1	2	3	4	5	6	7	8	Total
[M + Na]^+^	380	544	350	364	421	675	451	464	
Control	529.41 ± 5.24 ^a^	560.91 ± 21.74 ^a^	3.54 ± 0.00	320.62 ± 1.79 ^a^	tr	1191.66 ± 16.13 ^a^	22.01 ± 1.51 ^a^	889.05 ± 4.51 ^a^	3517.20 ± 50.92 ^a^
Substrate *	421.63 ± 7.94 ^b^	410.55 ± 0.18 ^b^	tr	1110.86 ± 11.97 ^b^	tr	1367.48 ± 6.72 ^b^	31.02 ± 0.24 ^b^	927.77 ± 7.36 ^b^	4269.31 ± 34.41 ^b^
Fly ash *	296.42 ± 1.16 ^c^	437.12 ± 15.95 ^c^	tr	252.41 ± 0.45 ^c^	tr	563.44 ± 0.67 ^c^	9.59 ± 0.09 ^c^	986.73 ± 0.73 ^c^	2545.71 ± 19.05 ^c^
Egg shells *	675.59 ± 12.81 ^d^	624.30 ± 1.63 ^d^	tr	482.70 ± 0.37 ^d^	tr	885.15 ± 5.21 ^d^	18.42 ± 1.47 ^d^	1093.87 ± 4.54 ^d^	3780.03 ± 26.03 ^d^
NaX *	537.29 ± 1.62 ^a^	307.61 ± 2.51 ^e^	tr	604.96 ± 0.11 ^e^	tr	1176.03 ± 2.85 ^e^	21.78 ± 0.05 ^a^	567.23 ± 7.05 ^e^	3214.90 ± 14.19 ^e^
CuSO_4_ solution	455.36 ± 3.90 ^e^	332.83 ± 6.52 ^f^	tr	593.34 ± 8.20 ^f^	tr	651.65 ± 7.38 ^f^	16.51 ± 0.14 ^e^	649.60 ± 0.94 ^f^	2699.29 ± 27.08 ^f^

* Control—plant cultivated in pure Compo Sana substrate and watered with tap water; * Compo Sana substrate mixed with various adsorbents cf. Table 1 and watered with tap water; CuSO_4_ solution—plant cultivated in pure Compo Sana substrate and watered with copper(II) sulfate solution; tr < 0.1 μmol/g DW; DW—dry weight; [M + Na]^+^, sodium adduct of desulfoglucosinolate; **1**—glucoraphanin; **2**—diglucothiobeinin; **3**—glucoibervirin; **4**—glucoerucin; **5**—4-methoxyglucobrassicin; **6**—dimeric 4-mercaptobutyl GSL; **7**—1,4-dimethoxyglucobrassicin; **8**—glucocamelinin. Chromatograms were given in Figure S1. Results are presented as mean ± SD (n = 3). Different superscript letters ^a−f^ in the same column denote statistically significant difference (*p* < 0.05) between samples.

## Supplementary Materials

The following supporting information can be downloaded online. Figure S1: Chromatograms of desulfoglucosinolates obtained from the rocket: d1—glucoraphanin; d2—4-(*β*-d-glucopyranosyldisulfanyl)butyl GSL; d3—glucoibervirin; d4—glucoerucin; d5—4-methoxyglucobrassicin; d6—dimeric 4-mercaptobutyl GSL; d7—1,4-dimethoxyglucobrassicin; d8—glucocamelinin (cf. Table 3 and Figure 2).

## Sample Availability

Samples of the compounds are not available from the authors.
