# Supplementary material for: Rocket (Eruca vesicaria (L.) Cav.) vs. Copper: The Dose Makes the Poison?
Source: Molecules. 2022 Jan 21;27(3):711. doi: 10.3390/molecules27030711 (PMC8838321; doi:10.3390/molecules27030711)
Supplement: Supplementary file 1 [file molecules-27-00711-s001.zip › molecules-1544704-supplementary.pdf]

molecules

Supplementary Materials

Rocket (*Eruca vesicaria* (L.) Cav.) vs. Copper: The Dose Makes the Poison?

Mario Nikola Mužek [1,*], Franko Burčul [2,*], Dario Omanović [3], Azra Đulović [4], Sandra Svilović [5] and Ivica Blažević [4]

[1] Department of Inorganic Technology, Faculty of Chemistry and Technology University of Split, Ruđera Boškovića 35, 21000 Split, Croatia, muky@ktf-split.hr
[2] Department of Analytical Chemistry, Faculty of Chemistry and Technology University of Split, Ruđera Boškovića 35, 21000 Split, Croatia; franko@ktf-split.hr
[3] Division for Marine and Environmental Research, Laboratory for physical chemistry of traces, Ruđer Bošković Institute, Bijenička cesta 54, 10000 Zagreb, Croatia; omanovic@irb.hr
[4] Department of Organic Chemistry, Faculty of Chemistry and Technology University of Split, Ruđera Boškovića 35, 21000 Split, Croatia; azra@ktf-split.hr (A.Đ.); blazevic@ktf-split.hr (I.B.)
[5] Department of Chemical Engineering, Faculty of Chemistry and Technology University of Split, Ruđera Boškovića 35, 21000 Split, Croatia; sandra@ktf-split.hr
* Correspondence: muky@ktf-split.hr (M.N.M.); franko@ktf-split.hr (F.B.)

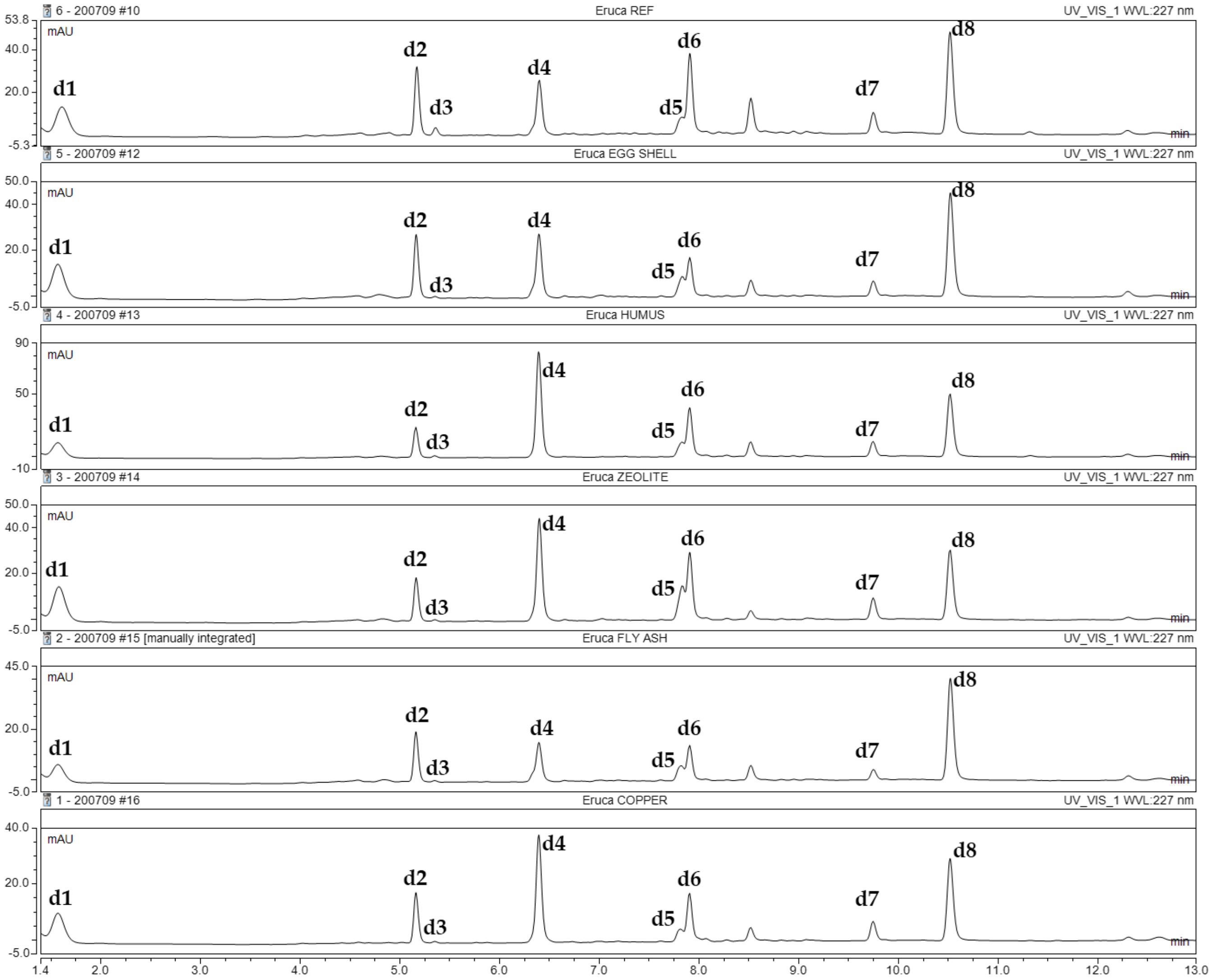

Figure S1. Chromatograms of desulfoglucosinolates obtained from the rocket (*Eruca vesicaria* (L.) Cav.): **d1** - glucoraphanin; **d2** - 4-(*β*-D-glucopyranosyldisulfanyl)butyl GSL; **d3** - glucoibervirin; **d4** - glucoerucin; **d5** - 4-methoxyglucobrassicin; **d6** - dimeric 4-mercaptobutyl GSL; **d7** - 1,4-dimethoxyglucobrassicin; **d8** - glucocamelinin (*cf.* Table 3 and Figure 2).
